# Nearly Complete Genome Sequences of Two Bovine Viral Diarrhea Virus Isolates, Subtype 1f Strain SLO/1170/2000 and Subtype 1d Strain SLO/2416/2002

**DOI:** 10.1128/MRA.00931-19

**Published:** 2019-11-14

**Authors:** Ivan Toplak, Danijela Rihtarič, Jože Grom, Nataša Toplak, Urška Kuhar

**Affiliations:** a Institute of Microbiology and Parasitology, Veterinary Faculty, University of Ljubljana, Ljubljana, Slovenia; b Omega d.o.o., Ljubljana, Slovenia; KU Leuven

## Abstract

Bovine viral diarrhea virus (BVDV) subtypes 1f and 1d were isolated for the first time in Slovenia in 1999 and detected later in a majority of BVDV-infected cattle herds. Here, we report the first nearly complete genome sequences of noncytopathogenic BVDV-1f strain SLO/1170/2000 and cytopathogenic BVDV-1d strain SLO/2416/2002, isolated in Slovenia.

## ANNOUNCEMENT

Bovine viral diarrhea virus (BVDV) is a widespread pathogen of ruminants that generates significant economic losses during acute and persistent infections (1, 2). Strains of BVDV are divided into two species, *Pestivirus A* (BVDV-1) and *Pestivirus B* (BVDV-2), within the *Pestivirus* genus of the family *Flaviviridae* (3). BVDV-1 is very heterogeneous and is further divided into 21 subgenotypes (4). The genomic RNA contains about 12,000 nucleotides (nt), with a single large open reading frame (ORF) encoding a polyprotein of about 3,900 amino acids (aa) that is preceded by a 5′-untranslated region (5′ UTR) of 370 to 385 nt and followed by a 3′ UTR of 185 to 273 nt. The gene order is 5′-N^pro^-C-E^rns^-E1-E2-p7-NS2-3(NS2-NS3)-NS4A-NS4B-NS5A-NS5B-3′ (5). Monitoring for BVDV infections was implemented in Slovenia in 1994 (6). Genetic typing of BVDV strains revealed that four subgenotypes of BVDV (1b, 1d, 1f, and 1g) were circulating in infected cattle farms between 1997 and 2004, with dominance of the subgenotypes 1f and 1d (7). Until now, only a single complete genome of BVDV-1e from Slovenia was published (8).

BVDV strain SLO/1170/2000 was isolated in 2000 from a serum sample collected from clinically healthy persistently infected cattle that produced no cytopathic effect on bovine turbinate (BT) cells, while BVDV strain SLO/2416/2002 was obtained from lymph nodes of a 22-month-old dead cow that suffered with mucosal disease and produced cytopathic effect on BT cells.

Total RNA extraction from supernatants of isolates was performed using the QIAamp viral RNA kit (Qiagen, Hilden, Germany). For sequencing of the BVDV strain SLO/1170/2000 genome, a previously described protocol was used (8). For sequencing of the BVDV strain SLO/2416/2002 genome, the cDNA was synthesized with the cDNA synthesis system (Roche, Manheim, Germany) and fragmented using the Covaris M220 instrument, targeting peak fragment lengths of 400 bp. The library was prepared with the GeneRead DNA Library L core kit (Qiagen) and quantified with a GeneRead Library Quant kit (Qiagen) and a Qubit v.3.0 fluorometer (Thermo Fisher Scientific, Carlsbad, CA, USA). Sequencing of both genomes was performed on the Ion PGM platform. Sequenced reads were quality checked and trimmed using Ion Torrent Suite and assembled into contigs by *de novo* assembly using Genome Sequencer software v.2.9 (Roche) with default parameters. To eliminate assembly errors, all sequenced reads were mapped against the assembled genomes with the Geneious reference mapper (Geneious software suite v.9.1.8; Biomatters Ltd., Auckland, New Zealand) with default parameters. Open reading frames (ORFs) were predicted with the Geneious ORF finder.

In total, 26,624 reads with a mean read length of 159 nt (for BVDV strain SLO/1170/2000) and 246,120 reads with a mean read length of 206 nt (for SLO/2416/2002) were obtained, and the assembled genomes had an average read coverage of 225× and 63×, respectively. The nearly complete genomes of SLO/1170/2000 and SLO/2416/2002 were 12,259 and 12,241 nt long with G+C contents of 46.8% and 45.1%, respectively. The single ORF on each genome was 3,898 aa long, and the two ORFs showed 87.7% aa similarity to each other. Compared to the complete sequences of BVDV available in GenBank (using NCBI blastn), strain SLO/1170/2000 displayed only 80.13% nucleotide similarity to strain SLO/2407/2006 (GenBank accession number KX577637) from Slovenia, belonging to the subgenotype BVDV-1e, whereas SLO/2416/2002 showed 90.40% nucleotide similarity to BVDV-1d strain BJ1308 from China (9). This study provides the nearly complete genome sequences of BVDV strains of the subtypes 1f and 1d circulating in Slovenia, which will contribute to a better understanding of the diversity and epidemiology of BVDV strains.

### Data availability.

The complete genome sequences of these two BVDV isolates, SLO/1170/2000 and SLO/2416/2002, have been deposited in GenBank under the accession numbers KX987157 and KY849592, respectively. The raw sequence data were deposited under BioProject number PRJNA576600.
